# The Ebola Virus Nucleoprotein Recruits the Nuclear RNA Export Factor NXF1 into Inclusion Bodies to Facilitate Viral Protein Expression

**DOI:** 10.3390/cells9010187

**Published:** 2020-01-11

**Authors:** Lisa Wendt, Janine Brandt, Bianca S. Bodmer, Sven Reiche, Marie Luisa Schmidt, Shelby Traeger, Thomas Hoenen

**Affiliations:** 1 Institute of Molecular Virology and Cell Biology, Friedrich-Loeffler-Institut, 17493 Greifswald, Germany; lisa.wendt@fli.de (L.W.); janine.brandt@fli.de (J.B.); bianca.bodmer@fli.de (B.S.B.);; 2 Department of Experimental Animal Facilities and Biorisk Management, Friedrich-Loeffler-Institut, 17493 Greifswald, Germany; sven.reiche@fli.de; 3 Laboratory of Virology, Division of Intramural Research, National Institute for Allergy and Infectious Diseases, National Institutes of Health, Hamilton, MT 59840, USA

**Keywords:** Ebola virus, filovirus, inclusion bodies, NXF1, liquid organelles, mRNA export

## Abstract

Ebola virus (EBOV) causes severe outbreaks of viral hemorrhagic fever in humans. While virus-host interactions are promising targets for antivirals, there is only limited knowledge regarding the interactions of EBOV with cellular host factors. Recently, we performed a genome-wide siRNA screen that identified the nuclear RNA export factor 1 (NXF1) as an important host factor for the EBOV life cycle. NXF1 is a major component of the nuclear mRNA export pathway that is usurped by many viruses whose life cycles include nuclear stages. However, the role of NXF1 in the life cycle of EBOV, a virus replicating in cytoplasmic inclusion bodies, remains unknown. In order to better understand the role of NXF1 in the EBOV life cycle, we performed a combination of co-immunoprecipitation and double immunofluorescence assays to characterize the interactions of NXF1 with viral proteins and RNAs. Additionally, using siRNA-mediated knockdown of NXF1 together with functional assays, we analyzed the role of NXF1 in individual aspects of the virus life cycle. With this approach we identified the EBOV nucleoprotein (NP) as a viral interaction partner of NXF1. Further studies revealed that NP interacts with the RNA-binding domain of NXF1 and competes with RNA for this interaction. Co-localization studies showed that RNA binding-deficient, but not wildtype NXF1, accumulates in NP-derived inclusion bodies, and knockdown experiments demonstrated that NXF1 is necessary for viral protein expression, but not for viral RNA synthesis. Finally, our results showed that NXF1 interacts with viral mRNAs, but not with viral genomic RNAs. Based on these results we suggest a model whereby NXF1 is recruited into inclusion bodies to promote the export of viral mRNA:NXF1 complexes from these sites. This would represent a novel function for NXF1 in the life cycle of cytoplasmically replicating viruses, and may provide a basis for new therapeutic approaches against EBOV, and possibly other emerging viruses.

## 1. Introduction

Ebola virus (EBOV) belongs to the genus *Ebolavirus* within the family *Filoviridae* and causes a severe hemorrhagic fever, called Ebola virus disease, in humans with high case fatality rates of about 40–60% [1,2]. Ongoing and past outbreaks of Ebola virus disease in Africa highlight the importance of a better understanding of the EBOV life cycle in order to develop new therapeutic approaches. During the viral life cycle the EBOV nucleoprotein (NP) encapsidates the negative-stranded RNA genome and is essential for viral replication and transcription [3]. NP interacts with the transcriptional activator viral protein 30 (VP30), which bridges NP and the RNA-dependent RNA polymerase L [4,5,6]. Furthermore, NP interacts with the polymerase cofactor VP35 [5,6]. This interaction regulates the oligomerization and RNA-binding of NP, and also bridges NP to L [5,6,7,8,9]. NP, VP35, VP30, and L, together with the RNA genome, form the ribonucleoprotein complex (RNP) and are sufficient to mediate viral replication and transcription [3], which takes place in cytoplasmic inclusion bodies [10]. The formation of these inclusion bodies is driven by expression of NP, which is localized in these structures not only during infection, but also after sole expression of this protein [5,6]. However, only limited knowledge exists regarding host factors that interact with the viral proteins and RNAs found in these structures. One such host factor that has been identified is importin-α7, which seems to be involved in inclusion body formation [11]. Marburg virus, a close relative of EBOV, was shown to recruit components of the endosomal sorting complex required for transport (ESCRT) to inclusion bodies to facilitate the trafficking of nucleocapsids to the plasma membrane for viral assembly and budding [12,13]. Kinases and phosphatases such as PP2A-B56 are also known to be recruited to inclusion bodies, and are important in regulating the activity of VP30 in viral RNA synthesis, which is dependent on its phosphorylation status [14,15]. Similarly, RBBP6 appears to regulate the balance of replication and transcription by binding to VP30, and also Staufen1 was described to influence viral RNA synthesis [16,17]. Finally, EBOV VP35 appears to sequester cellular stress granule proteins within inclusion bodies in order to prevent stress granule formation [18].

To obtain a comprehensive picture of the pro- and anti-viral factors that are important for EBOV RNA synthesis (i.e., genome replication and transcription) and/or protein expression, we recently performed a genome-wide siRNA screen [19]. As primary readout we used a minigenome assay (reviewed in [20]). In this assay RNA minigenomes, i.e., miniature versions of the EBOV genome with all viral genes removed and replaced with a reporter gene, are expressed in mammalian cells together with the viral RNP proteins. Since the minigenomes still contain the regulatory terminal leader and trailer regions of the EBOV genome that carry the replication and transcription promoters, the RNP proteins recognize these minigenomes as authentic templates. This results in their replication and transcription, and ultimately reporter protein expression reflecting these aspects of the viral life cycle. In our siRNA screen we showed that the nuclear RNA export factor 1 (NXF1) is necessary for the EBOV life cycle, and also for the life cycle of the highly pathogenic New World arenavirus Junín virus. These data suggest a mechanism of action that may be conserved among several cytoplasmically replicating negative-stranded RNA viruses (NSVs) that share commonalties in their replication cycles, such as replication in cytoplasmic inclusion bodies [10,21,22,23,24]. Importantly, we also could show that NXF1 is important for the life cycle of EBOV in context of infectious virus [19]. However, the precise function of NXF1 in the EBOV life cycle remained unclear.

NXF1 is a crucial component of the nuclear mRNA export pathway, where it exports cellular mRNAs from the nucleus by interacting with nucleoporins [25,26]. For this interaction NXF1 forms a stable heterodimer with p15 (also called NXT1) via its NTF2-like domain (NTF2) [27,28]. The NTF2-like domain is one of the five functional domains found in NXF1, with the other ones being the RNA-binding domain (RBD), the pseudo RNA recognition motif (RRM), a region of leucine-rich repeats (LRR), and the ubiquitin-associated domain (UBA) [27,29,30]. NTF2 and UBA promote interaction with nucleoporins, while the three amino-terminal domains (RBD, RRM, and LRR) are important for mRNA binding [26,27,31]. Recently, NXF1 was shown to be loaded onto cellular mRNAs co-transcriptionally, a process that depends on several other export adapters that promote the interaction between NXF1 and mRNA, in order to enable mRNA export from the nucleus [29,31,32]. However, the NXF1-driven nuclear mRNA export pathway is also utilized by many viruses that replicate in the nucleus [33,34,35,36]. For instance, herpesviruses and influenza viruses usurp this host pathway to export their viral RNAs, and particularly their mRNAs, from the nucleus [33,34,37,38,39]. Furthermore, NXF1 is known to export retroviral RNAs containing constitutive transport elements and Hepatitis B virus pregenomic RNA [35,36]. Thus far, the only non-nuclear function of NXF1 has been described for gammaretroviruses, which use NXF1 for loading of gag RNA onto polysomes, rather than for mRNA export [40]. However, since EBOV replicates in cytoplasmic inclusion bodies, the function of NXF1 during an EBOV infection must differ from those previously described [10]. Therefore, we characterized the interaction of NXF1 with EBOV on a biochemical and functional level. Based on our results we propose a model in which NXF1 is recruited to inclusion bodies where it interacts with NP and subsequently with viral mRNAs, which it then exports from inclusion bodies.

## 2. Materials and Methods

### 2.1. Cells

HEK 293T (human embryonic kidney) cells (Collection of Cell Lines in Veterinary Medicine CCLV-RIE 1018) and Huh7 (human hepatocarcinoma) cells (kindly provided by Stephan Becker, Philipps University Marburg, Marburg, Germany) were maintained in Dulbecco’s modified Eagle’s medium (DMEM; ThermoFisher Scientific, Waltham, MA, USA) supplemented with 10% fetal bovine serum (FBS), 100 U/mL penicillin and 100 µg/mL streptomycin (PS; ThermoFisher Scientific) and 1× GlutaMAX (ThermoFisher Scientific). All cells were grown at 37 °C with 5% CO_2_.

### 2.2. Plasmids and Antibodies

Expression plasmids for the RNP proteins, the T7 polymerase and the T7-driven monocistronic minigenomes (pT7-1cis-vRNA-rLuc, pT7-1cis-vRNA-nLuc) have been previously described [19,41]. Plasmids for the expression of N-terminally flag/HA-tagged constructs were generated by insertion of the respective genes (NXF1, p15, NP) into a pCAGGS-flag/HA vector. All mutant versions and individual domains of NXF1 were first inserted into pCAGGS and subsequently subcloned into the pCAGGS-flag/HA plasmid. EBOV VP40 and VP24 were also cloned into pCAGGS. The PolII-driven replication-deficient minigenome expressing NanoLuc luciferase as its reporter was generated by flanking the minigenome in vRNA-orientation with hammerhead and Hepatitis Delta Virus ribozymes and insertion of the minigenome into pCAGGS. Deletion of 55 nt in the antigenomic replication promotor to generate a replication-deficient version of the PolII-driven minigenome was performed as previously described [41]. Detailed cloning strategies are available on request.

The anti-flag (mouse anti-flag mAb, clone M2, cat. no. F1804) antibody used for co-immunoprecipitation assays (coIPs) and Western blot analyses was purchased from Sigma-Aldrich. Primary antibodies against NP (rabbit anti-EBOV NP pAb, cat. no. 0301-012), VP35 (rabbit anti-ZEBOV VP35 affinity purified polyclonal antibody, cat. no. 0301-040) and VP40 (mouse anti-EBOV VP40 mAb, clone 3G5, cat. no. 0201-016) were obtained from IBT Bioservices, and the antibody against NXF1 (mouse anti-NXF1 mAb, clone 53H8, cat. no. ab50609) was obtained from Abcam. For VP30, a mouse monoclonal antibody (clone 5F11 A1) was produced by the FLI biobank as previously described [42] and following approved protocols using affinity-purified flag/HA-tagged VP30 expressed in mammalian cells as the immunogen. Secondary antibodies against mouse (Peroxidase AffiniPure Goat Anti-Mouse IgG, light chain specific, cat. no. 115-035-174, used for Western blots with exception of that shown in Figure 4) and rabbit (Peroxidase AffiniPure Goat Anti-Rabbit IgG H+L, cat. no. 111-035-003) were obtained from Jackson Immunoresearch or Abcam (anti-mouse IgG for IP, HRP, cat. no. ab131368, used for the Western blot shown in Figure 4).

### 2.3. Co-Immunoprecipitation of Viral Proteins

**Figure 1:**
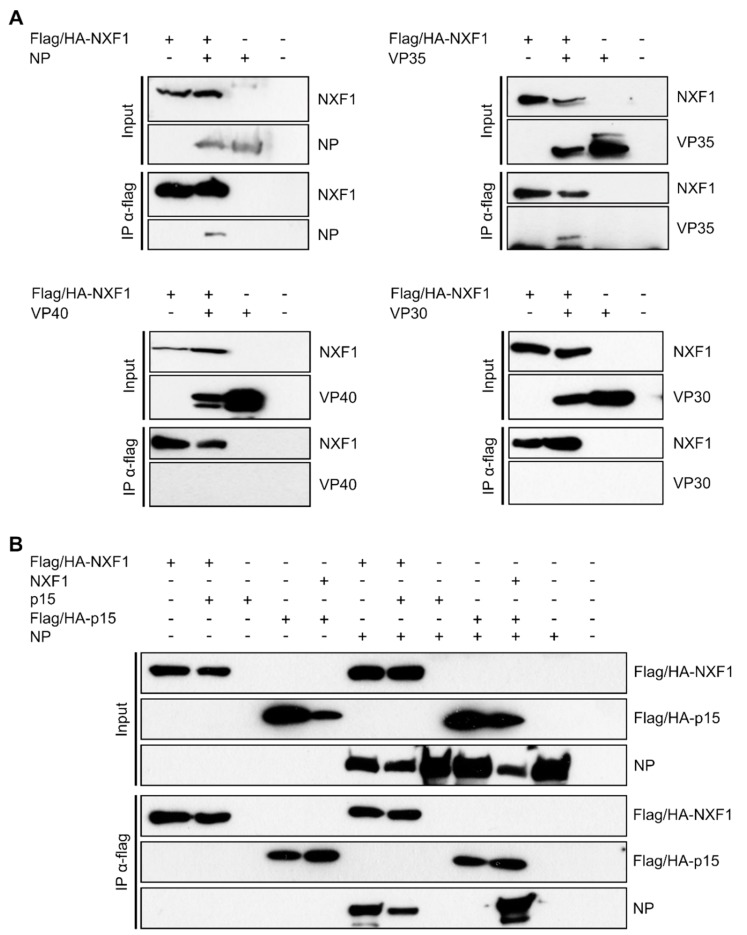
Interaction of NXF1 with nucleoprotein (NP) and VP35. (**A**) Immunoprecipitation of NXF1 and coIP of viral proteins. 293T cells were transfected with the plasmids encoding for flag/HA-NXF1 and one of the viral proteins NP, VP35, VP40 or VP30. Forty-eight hours p.t. flag/HA-NXF1 was precipitated using anti-flag antibodies, and input and precipitates were analyzed via SDS-PAGE and subsequent Western blot. NXF1 was detected with anti-flag antibodies, and anti-NP, anti-VP35, anti-VP40 or anti-VP30 antibodies were used for the detection of the respective viral protein. In case of the VP35 blot, the band at the lower edge of the blot that is partially cut off is caused by the light chain of the IP antibody. (**B**) Influence of p15 on the interaction between NXF1 and NP. Flag/HA-tagged NXF1 or p15 was precipitated from transfected 293T cells using anti-flag antibodies. Input and precipitate samples were subjected to SDS-PAGE and analyzed by Western blot using anti-flag antibodies for the detection of p15 and NXF1, whereas an anti-NP antibody was used for the detection of NP.

**Figure 2:**
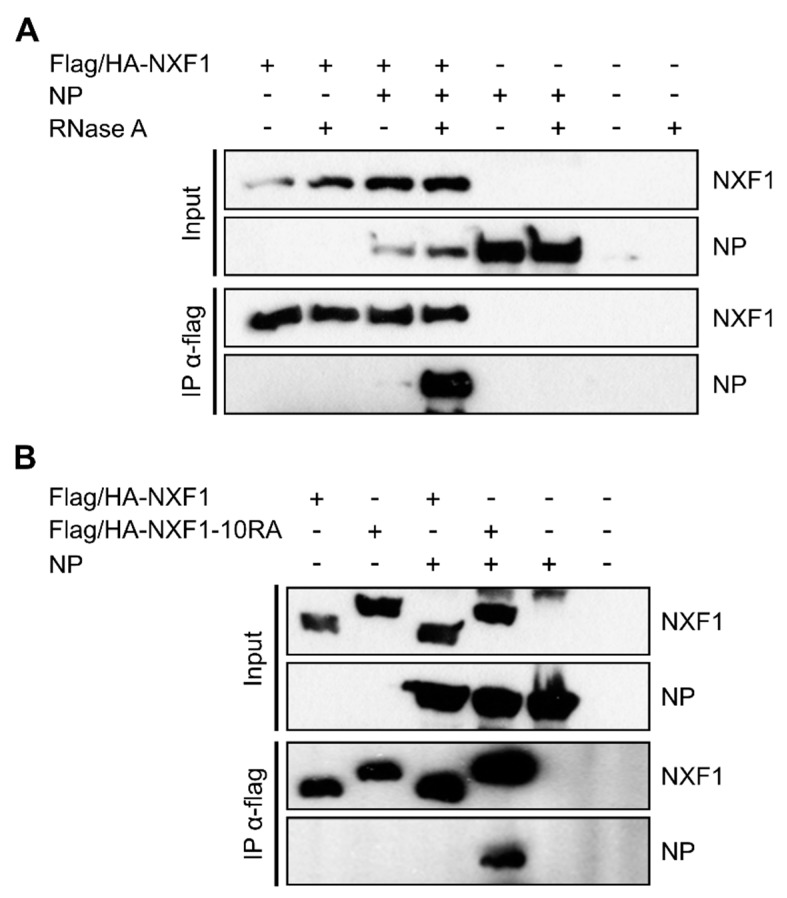
RNA dependence of the interaction between NXF1 and NP. (**A**) Absence of single-stranded RNA enhances interaction between NP and NXF1. 293T cell lysates containing overexpressed flag/HA-NXF1 and NP were treated with RNase A (100 µg/mL) or remained untreated before flag/HA-NXF1 was precipitated using anti-flag antibodies. Input and precipitates were analyzed via SDS-PAGE and Western blot using anti-flag and anti-NP antibodies. (**B**) RNA binding-deficient NXF1-10RA displays a stronger interaction with NP than wildtype NXF1. 293T cells were transfected with plasmids encoding for either flag/HA-NXF1 or flag/HA-NXF1-10RA together with NP, and 48 h p.t. cells were lysed and subjected to coIP analyses. Pulldowns were performed using anti-flag antibodies, and precipitates as well as input samples were analyzed via SDS-PAGE and Western blot using anti-flag and anti-NP antibodies.

For coIPs, 293T cells were seeded into 6-well plates and transfected at 30% confluency with expression plasmids encoding the flag/HA-tagged host proteins (NXF1, p15, mutated versions or individual domains of NXF1) and for their putative interaction partners (p15, NXF1 or EBOV proteins) using Transit LT-1 (Mirus Bio LLC, Madison, WI, USA) following the manufacturer’s instructions. Supernatant was exchanged after 24 h and coIP was performed 48 h post transfection (p.t.). To this end, cells were washed in PBS and pelleted before being lysed in 1 mL coIP lysis buffer (1% NP-40; 50 mM Tris; 150 mM NaCl; pH 7.4) with protease inhibitor (cOmplete; Roche, Basel, Switzerland) and incubated with rotation at 15 rpm for 2 h at 4 °C. Initial experiments investigating an interaction between viral proteins and NXF1 (Figure 1A) where performed in the absence of RNase treatment. However, since RNase treatment increases the interaction between NXF1 and NP (see Figure 2), in all further experiments 100 µg/mL RNase A (Machery-Nagel, Düren, Germany) was added to the samples prior to incubation. Lysates were separated from cell debris by centrifugation (11,000× *g*, 10 min, 4 °C) and 150 µL was used for the input control (representing a sixth of the whole lysate and 20% of the sample subjected to IP) and subjected to acetone precipitation. To this end 750 µL acetone pre-chilled to −20 °C was added to the samples, and they were incubated for at least 30 min at −20 °C. Proteins were then pelleted by centrifugation (20,000× *g*, 15 min, 4 °C), resuspended in 60 µL 1× SDS sample buffer and boiled for 10 min at 99 °C. The remaining 750 µL of lysate was added to the prepared bead-antibody solution (Dynabeads Protein G, ThermoFisher Scientific; 1 µL anti-flag M2 antibody per 10 µL beads), resuspended, and the IP was performed for 10 min (as recommended by the manufacturer to minimize non-specific binding) at room temperature with rotation at 15 rpm. Beads were washed three times with PBS containing 0.02% Tween-20 and transferred to new tubes. The beads were resuspended in 60 µL 1× SDS sample buffer and boiled for 10 min at 99 °C. Input and coIP samples were analyzed via SDS-PAGE and semi-dry Western blot, using an ethanol-containing blotting buffer (5.8 g Tris(hydroxymethyl)aminomethan; 2.9 g Glycin; 200 mL Ethanol; ad 1 mL H_2_O) together with Nitrocellulose membranes and a blotting current of 0.8 mA/cm^2^ for 1 to 2 h, and 7% skim milk powder in PBS for blocking.

### 2.4. Immunofluorescence Analysis

Huh7 cells were seeded onto coverslips in 12-well plates and transfected the next day with 500 ng pCAGGS-EBOV-NP, 500 ng pCAGGS-p15 and 500 ng pCAGGS-flag/HA-NXF1 (or pCAGGS-flag/HA-NXF1-10RA or pCAGGS-flag/HA-NXF1-ΔRBD) using Polyethylenimine (Sigma-Aldrich) following the manufacturer’s instructions, or remained untransfected (mock). Forty-eight hours p.t., cells were fixed using 4% PFA in DMEM, treated with 0.1 M glycine and permeabilized with 0.1% Triton X-100 (in PBS++ (PBS with 1 mM Ca^2+^ and 1 mM Mg^2+^) with 10% fetal calf serum). Primary antibodies (rabbit anti-EBOV-NP 1:500; mouse anti-flag 1:2000) were diluted in PBS with 10% fetal calf serum, and cells were incubated for 1 h at 4 °C with the antibody solutions. The same procedure was used for the secondary antibodies (Alexa Fluor-568 anti-rabbit 1:1500, Alexa Fluor-488 anti-mouse 1:1200; both ThermoFisher Scientific). After staining, cells were washed with PBS and water before mounting with ProLong Diamond Antifade mountant with DAPI (ThermoFisher Scientific). Slides were analyzed by confocal laser scanning microscopy using a Leica SP5.

### 2.5. siRNA Knockdown and Minigenome Assay

293T cells were reverse transfected with 12 pmol pre-designed silencer select siRNAs (NXF1 #1: s20532; NXF1 #2: s20533; Negative Control siRNA No. 2; all ThermoFisher Scientific) or an anti-L siRNA (5’-UUU AUA UAC AGC UUC GUA CUU-3’) using Lipofectamine RNAiMax (ThermoFisher Scientific) following the manufacturer’s instructions in 12-well plates. Forty-eight hours p.t., cells were transfected with all plasmids for the respective minigenome assay using Transit LT-1 as described before [41]. For the classical T7-driven replication-competent minigenome system (including the analysis of mRNA levels), the cells were transfected with the plasmids encoding the T7-polymerase, the T7-driven monocistronic minigenome (1cis-vRNA-nLuc), the RNP proteins L, NP, VP30 and VP35 and firefly luciferase or green fluorescent protein (GFP) as a control. For the replication-deficient minigenome system, cells were transfected with the plasmids encoding the PolII-driven replication-deficient minigenome (pCAGGS-1cis-vRNA-nLuc-RdM), the RNP proteins L, NP, VP30 and VP35 and firefly luciferase as a control. A further 48 h later cells were harvested for either determination of reporter activity or RNA isolation (see below). For measuring the reporter activity, cells were lysed for 10 min in 1× Lysis Juice (PJK, Kleinblittersdorf, Germany) and lysates were cleared by centrifugation (3 min at 10,000× *g*, room temperature). Forty µL of cleared lysate was added to either 40 µL of Beetle Juice (PJK) or Nano-Glo Luciferase Assay Reagent (Promega, Madison, WI, USA) in opaque 96-well plates. Luminescence was measured using a Glomax Multi (Promega) microplate reader. Minigenome reporter activity was normalized to control firefly luciferase activity, which acts as a measure for plasmid-driven gene expression.

### 2.6. Infection Experiments

siRNA-transfected 293T cells in 12-well format were infected with 5000 TCID50 rgEBOV-luc2 [43] 24–48 h p.t. Twenty-four hours post infection (p.i.) cells were lysed in 300 µL GloLysis buffer (Promega) for 10 min and 50 µL samples were mixed with either 50 µL BrightGlo (Promega) or 50 µL CellTiterGlo (Promega) in white opaque 96-well plates and measured on a Tecan F200Pro. Firefly luciferase activity reflecting viral gene expression was normalized to luciferase activity in the CellTiterGlo assay (which quenches firefly luciferase activity and instead measures intracellular ATP levels as proxy for cell viability using an UltraGlo luciferase). For mRNA quantification, identically transfected and infected cells were harvested in 700 µL RLT buffer (Qiagen, Hilden, Germany), and after addition of 700 µL 70% EtOH and removal from the BSL4-laboratory RNA was extracted using the RNEasy kit (Qiagen) following the manufacturer’s instructions. mRNA quantification was performed as described below.

For immunofluorescence analysis, Huh7 cells in ibidi 8-well chamber slides were infected with rgEBOV-wt [44] at an MOI of 1. Sixteen hours p.i., cells were washed with PBS and fixed using 10% formalin. The subsequent procedure for immunofluorescence staining was performed as described above. All experiments involving infectious EBOV were done in the BSL4 laboratory of the Friedrich-Loeffler-Institut following approved SOPs.

### 2.7. RNA Co-Immunoprecipitation

For mRNA-coIPs 293T cells were transfected using Transit LT-1 (Mirus Bio LLC) following the manufacturer’s instructions with the plasmids encoding for flag/HA-NXF1 or its mutants (1500 ng; amounts per well), GFP (250 ng) as a transfection control, as well as all T7 monocistronic minigenome components (T7—250 ng, L—1000 ng, NP—125 ng, VP30—65 ng, VP35—125 ng, monocistronic EBOV minigenome encoding Renilla luciferase—250 ng), while as a control either L (-L) or flag/HA-NXF1 (-NXF1) was omitted. For vRNA-coIPs 293T cells were transfected with the plasmids encoding for the T7 polymerase (250 ng) and the T7-driven monocistronic minigenome encoding Renilla luciferase (250 ng) together with either flag/HA-NXF1 (1500 ng) or a combination of flag/HA-NP (1500 ng), VP35 (1250 ng) and VP24 (1250 ng). As controls, either the minigenome (-1cis) or the flag/HA-tagged protein (1cis w/o flag-protein) was omitted. The coIP procedure was then carried out 48 h p.t. as described above for the protein coIPs, but the beads were resuspended in 140 µL PBS instead of 1× SDS sample buffer and not boiled before they were subjected to RNA isolation and RT-qPCR.

### 2.8. RNA Isolation and RT-qPCR

RNA isolation from minigenome-transfected cell lysates was performed using the NucleoSpin RNA kit (Machery-Nagel), and RNA isolation from coIP precipitates was performed using the QIAmp Viral RNA Mini kit (Qiagen), both according to the manufacturers’ instructions. The beads were separated from the precipitate-buffer solution with the use of a magnetic rack prior to loading of the spin columns to avoid clogging of the columns. All RNA samples were treated with DNase (TURBO DNA-free kit; ThermoFisher Scientific) following the manufacturer’s instructions to avoid plasmid contamination. The resulting RNA samples were then used for the generation of cDNA with either oligo(dT)-primers for total mRNA or strand-specific primers for the reporter gene in vRNA-orientation (Renilla: luc(−) 5’-CCA AAC AAG CAC CCC AAT CA-3’) using RevertAid Reverse Transcriptase (ThermoFisher Scientific) following the manufacturer’s instructions. The subsequent qPCR was performed using the PowerUp SYBR Green Master Mix (ThermoFisher Scientific) with 1 µL of cDNA and primers targeting either the reporter genes (NanoLuc: nluc(−) 5’-TTC AGA ATC TCG GG GTG TCC-3’, nluc(+): 5’-CGT AAC CCC GTC GAT TAC CA-3’; Renilla: luc(−), luc(+) 5’-TGT GCC ACA TAT TGA GCC AG-3’; firefly: luc2(−) 5’-CAG TCG TCG TGC TGG AAC AC-3’, luc2(+) 5’-GTC CAA CTT GCC GGT CAG TC-3’), or as a control for the minigenome experiments GFP (GFP(+) 5’-CTT GTA CAG CTC GTC CAT GC-3’, GFP(−) 5’-CGA CAA CCA CTA CCT GAG CAC-3’) and for the infection experiments GAPDH (GAPDH(−) 5’-CCA GGT GGT CTC CTC TGA CTT CAA-3’, GAPDH(+) 5’-ATA CCA GGA AAT GAG CTT GAC A-3’). Values for luciferase reporter mRNA levels were normalized to GFP/GAPDH mRNA levels and values for the mRNA coIP samples were normalized to their respective -L controls.

### 2.9. Statistical Analyses

One-way ANOVA with Dunett’s multiple comparisons test (Figure 7A–E) or Sidak’s multiple comparisons test (Figure 7F–G) were performed using the GraphPad Prism 8 software.

## 3. Results & Discussion

### 3.1. NXF1 Interacts with NP and VP35

To assess whether NXF1 interacts with EBOV proteins, we performed co-immunoprecipitation (coIP) assays using overexpressed flag/HA-NXF1 in the presence of either NP, VP35, VP30, or the EBOV matrix protein VP40, to find a possible interaction partner (Figure 1A). Using this approach, we could co-precipitate NP as well as VP35 with NXF1 (Figure 1A, second lanes), indicating that NXF1 interacts with both viral proteins. In contrast, VP40 and VP30 could not be co-precipitated with NXF1, indicating that most likely there is no interaction between NXF1 and these viral factors. Since NXF1 is known to form stable heterodimers with p15 in order to promote the interaction of NXF1 with nucleoporins and thereby fulfill its cellular function in nuclear export, we also performed coIPs of NP with NXF1 in presence and absence of overexpressed p15 [25,28]. The amount of NP co-precipitated with NXF1 was not increased in presence of p15 (Figure 1B, sixth and seventh lane). Further, p15 alone failed to co-precipitate NP, while co-precipitation could be recovered by NXF1 overexpression (Figure 1B, ninth and 10th lane). These data show that p15 is dispensable for the interaction between NXF1 and NP, and that the formation of the NXF1:p15 heterodimer is not necessary to mediate an interaction with NP. This is in contrast to other viruses interacting with NXF1 such as Hepatitis B virus, where HBc was shown to interact with both NXF1 and p15 [35].

### 3.2. NP and Cellular RNAs Compete for Interaction with NXF1

Since NP, VP35, and NXF1 are all known to be RNA-binding proteins, we next analyzed the dependence of their interaction on RNA by treating samples with RNase A to digest single-stranded RNAs before they were used for coIPs [25,45,46]. In case of VP35 the interaction with NXF1 was clearly dependent on the presence of RNA (Figure S1), raising the possibility that the observed interaction is merely due to unspecific binding of the same RNA, so that we did not further investigate this interaction, but rather focused on the interaction between NP and NXF1. Here, to our surprise, the experiments revealed that the interaction between NP and NXF1 was stronger in the presence of RNase A (Figure 2A, third and fourth lane), indicating a negative impact of single-stranded RNA on their interaction. To confirm this result, we performed coIPs with a mutant version of NXF1 that has been described to have lost its RNA-binding capability due to an exchange of ten arginines to alanines within its RNA-binding domain [29,31]. Using this mutant (NXF1-10RA) we observed that much more NP was co-precipitated than when using wildtype NXF1 (Figure 2B, third and fourth lane), similar to the results seen after RNase A treatment. Indeed, it was not possible to find conditions where both the very strong band for NP co-precipitated by NXF1-10RA and the much weaker band for NP co-precipitated by wildtype NXF1 could be visualized together with reasonable band intensities. This suggests that the observed differences in NXF1:NP interaction in the presence/absence of RNA are linked to the RNA-binding activity of NXF1. The more prominent interaction between NXF1 and NP in the absence of RNA-binding by NXF1 further suggests that there is competition between NP and single-stranded RNA for the binding of NXF1. Finally, the strong interaction between NXF1-10RA and NP also shows that the amino acids within NXF1 that are important for its RNA-binding capability are dispensable for the interaction with NP.

### 3.3. Interaction with NP Is Mediated by the RNA-Binding Domain of NXF1

**Figure 3:**
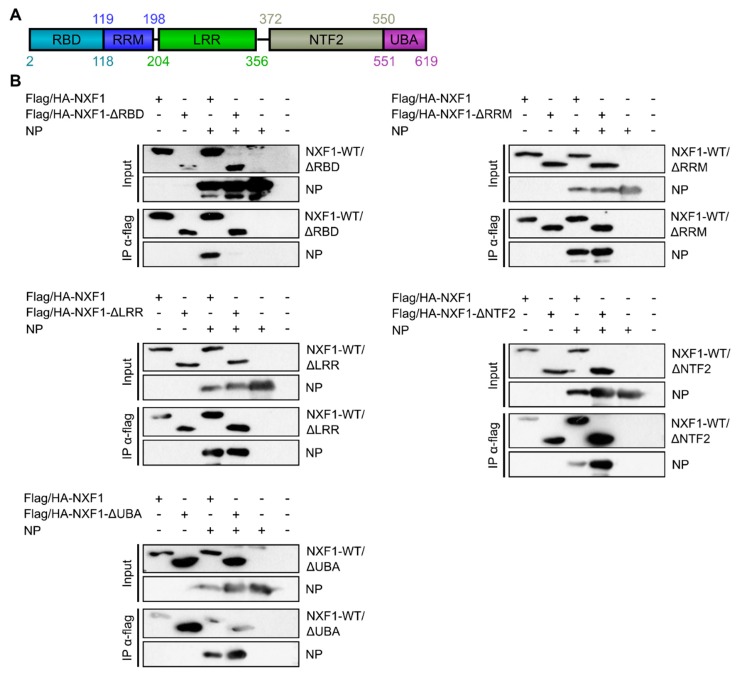
Identification of NXF1 domains necessary for the interaction with NP. (**A**) Domain organization of NXF1. Numbers indicate amino acid positions. (**B**) The RNA-binding domain of NXF1 is essential for its interaction with NP. 293T cells were transfected with plasmids encoding flag/HA-NXF1 or flag/HA-tagged NXF1 mutants together with NP. Forty-eight hours p.t., immunoprecipitation was performed using anti-flag antibodies. Subsequent SDS-PAGE and Western blot analyses with input and precipitates were performed using anti-flag and anti-NP antibodies.

To identify the domain of NXF1 that facilitates the interaction with NP, we generated different mutants of NXF1, each lacking one of its five described domains (Figure 3A) [29]. Using these mutants in coIP studies, we could co-precipitate NP with four of the five deletion mutants (ΔRRM, ΔLRR, ΔNTF2, ΔUBA), demonstrating that none of these domains is essential for the interaction (Figure 3B, fourth lanes). In contrast, NXF1-ΔRBD failed to co-precipitate NP, indicating that it is the RBD that is required for the interaction between these proteins.

**Figure 4:**
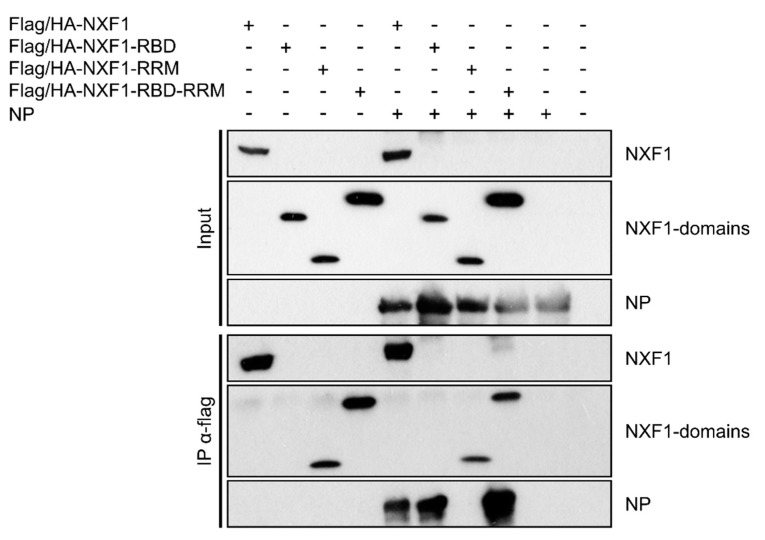
Role of the RNA-binding domain (RBD) and RNA recognition motif (RRM) in the interaction between NXF1 and NP. Immunoprecipitations of lysates from 293T cells transfected with flag/HA tagged NXF1, NXF1-RBD, NXF1-RRM or NXF1-RBD-RRM together with NP using anti-flag antibodies were performed 48 h p.t. Input and precipitate samples were subjected to SDS-PAGE and Western blot, and NXF1 and NP were detected using anti-flag and anti-NP antibodies, respectively.

To further analyze whether the RBD is also sufficient to mediate an interaction with NP, we also performed coIPs of NP with only the RBD of NXF1, as well as with NXF1-RRM and NXF1-RBD-RRM as controls. In these experiments, NP could be co-precipitated with the RBD and the RBD-RRM, but not with the RRM alone (Figure 4, sixth, eighth and seventh lane). This demonstrates that the RBD is not only required, but also sufficient for the interaction between NP and NXF1, while the RRM is neither required or sufficient for this interaction, nor does it interfere with it.

### 3.4. NXF1 Is Recruited into NP-Derived Inclusion Bodies

**Figure 5:**
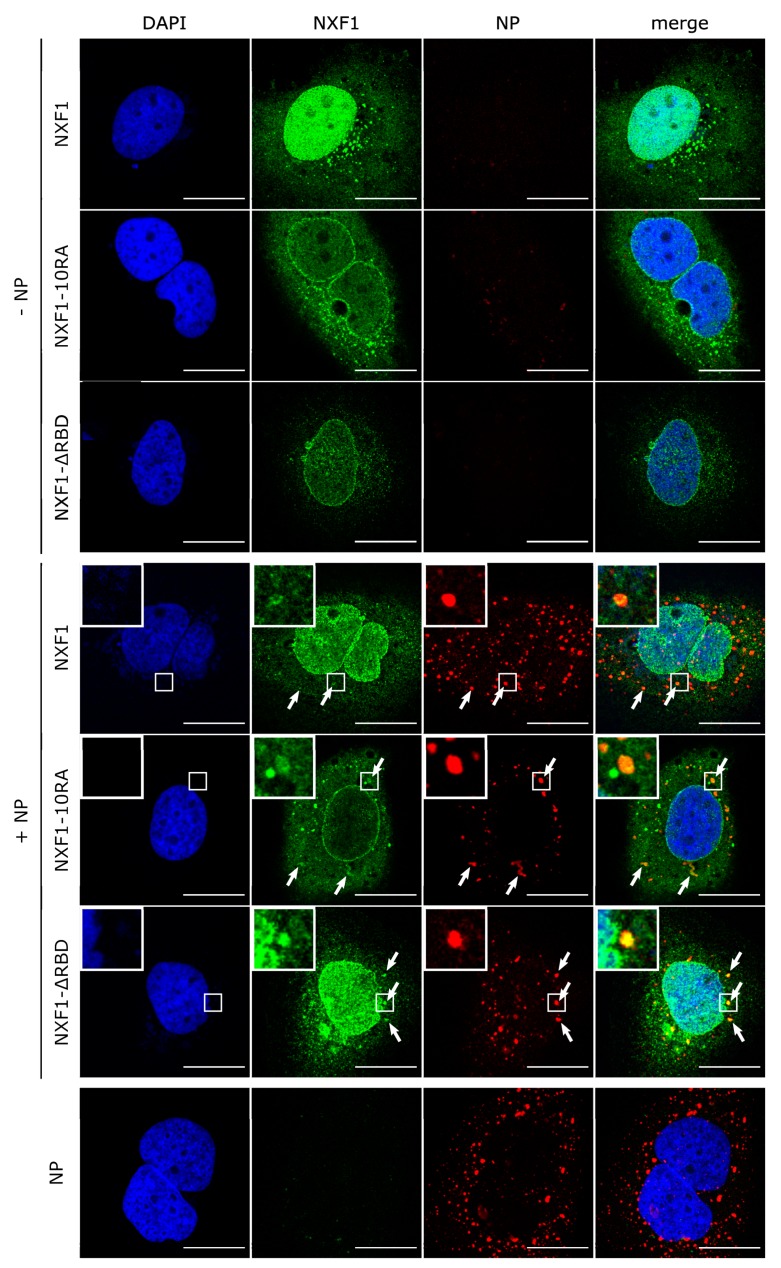
Recruitment of NXF1 into NP-derived inclusion bodies. Huh7 cells were transfected with plasmids encoding flag/HA-NXF1, flag/HA-NXF1-10RA or flag/HA-NXF1-ΔRBD together with NP as indicated. Forty-eight hours p.t., cells were fixed with 4% PFA and permeabilized with Triton X-100. NXF1 (shown in green) was detected with anti-flag antibodies and NP (shown in red) with anti-NP antibodies. Nuclei were stained with DAPI (shown in blue), and cells were visualized by confocal laser scanning microscopy. Scale bars indicate 20 µm. Arrows point out colocalization, and insets show magnifications of indicated areas.

Genome replication and transcription of EBOV is known to take place in inclusion bodies within the cytoplasm [10,14], and overexpression of NP alone leads to formation of such inclusion bodies [6]. In contrast, NXF1 is mainly localized in and around the nucleus [25,26]. Therefore, we wanted to analyze the influence of NP overexpression (and thus inclusion body formation) on the intracellular localization of NXF1. As previously shown [5,6], NP formed the typical inclusion bodies in the cytoplasm when expressed alone (Figure 5), and thus was used as a marker for these structures, while overexpression of NXF1 alone showed a localization in the nucleus and in the perinuclear region. This is in line with previous publications, which have described both a nuclear localization, and particularly a nuclear rim staining mediated by the NTF2 and UBA domains of NXF1 [27], as well as a cytoplasmic localization of NXF1, e.g., inside stress granules [47]. Co-expression of NP and wildtype NXF1 led to no dramatic changes in the intracellular distribution of NXF1, although small amounts of NXF1 could be detected in the NP-derived inclusion bodies. In contrast, the intracellular localization of NXF1-10RA changed upon co-expression of NXF1-10RA, and it clearly accumulated in NP-derived inclusion bodies. Similarly, NXF1-ΔRBD also accumulated in inclusion bodies, indicating that the accumulation of RNA binding-deficient NXF1 in inclusion bodies is not due to the identified direct interaction of NP with the RBD of NXF1, but that NXF1 is recruited via another mechanism. Importantly, while in the coIP experiments NXF1-10RA showed a stronger interaction with NP than wildtype NXF1, this was not the case for NXF1-ΔRBD, which is no longer able to interact with NP. Therefore, the fact that both of these NXF1 mutants accumulate in inclusion bodies suggests that the loss of their ability to bind RNA, but not changes to their ability to bind NP, dictates this phenotype. Together, these data suggest that NXF1 can enter inclusion bodies, but that only RNA binding-deficient NXF1 is retained and, therefore, accumulates in these structures, whereas RNA binding-competent NXF1 rapidly leaves them again.

**Figure 6:**
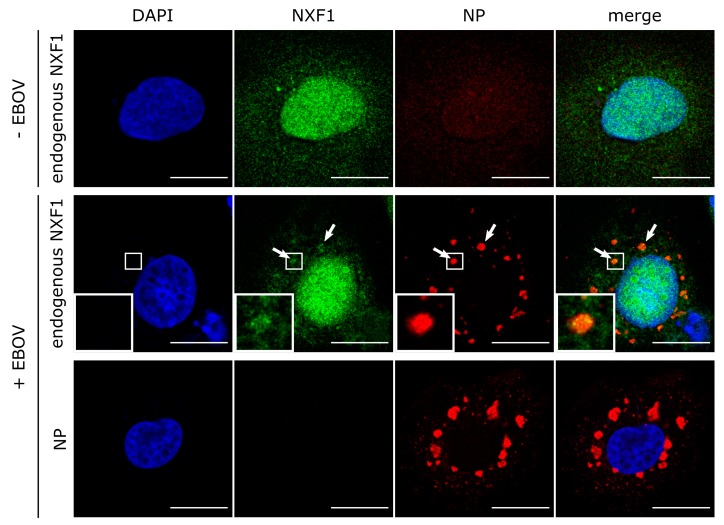
Recruitment of endogenous NXF1 into EBOV inclusion bodies. Huh7 cells were infected with rgEBOV. Sixteen hours p.i., cells were fixed with 10% formalin and permeabilized with Triton X-100. NXF1 (shown in green) was detected with anti-NXF1 antibodies and NP (shown in red) with anti-NP antibodies. Nuclei were stained with DAPI (shown in blue), and cells were visualized by confocal laser scanning microscopy. Scale bars indicate 20 µm. Arrows point out colocalization, and insets show magnifications of indicated areas.

To further substantiate these results, we analyzed the localization of endogenous NXF1 in context of infectious EBOV (Figure 6). As shown above with overexpressed NXF1, endogenous NXF1 was mostly localized in the nucleus. In contrast, NP was only detectable in the cytoplasmic inclusion bodies. Staining of both NXF1 and NP in the context of an EBOV infection showed a subtle but clearly visible relocalization of NXF1 into inclusion bodies, supporting the results obtained using overexpression of NP and NXF1.

### 3.5. NXF1 Influences Protein Expression but Not Viral mRNA Transcription

**Figure 7:**
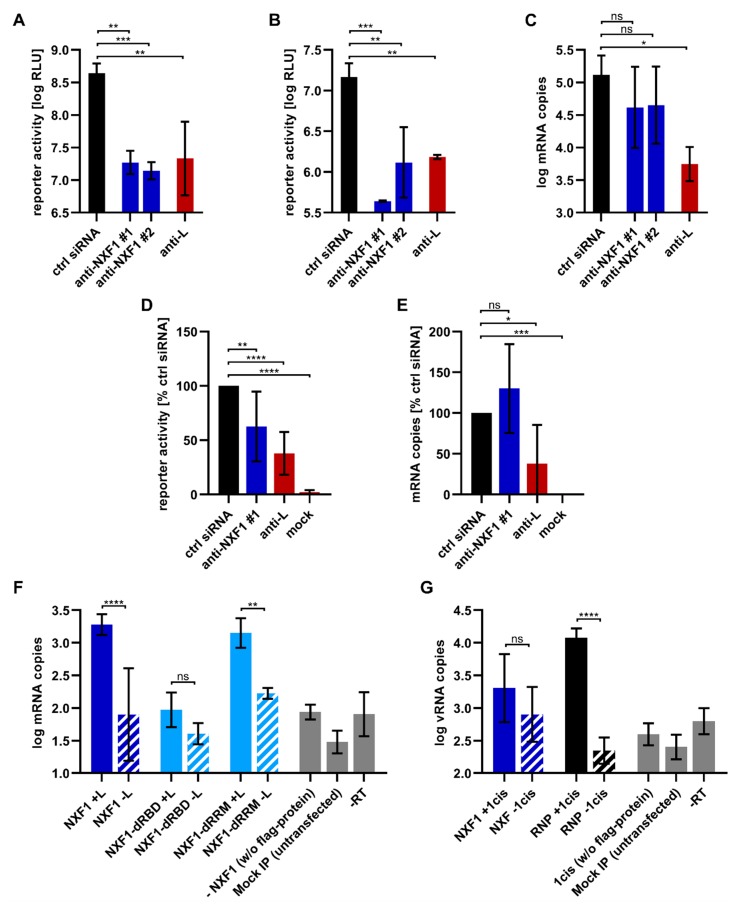
Influence of NXF1 on viral RNA synthesis and protein expression and interaction of NXF1 with viral RNAs. (**A**) Analysis of viral RNA synthesis and protein expression in NXF1 knockdown cells. 293T cells were transfected with siRNAs targeting either NXF1 (anti-NXF1) or L (anti-L) or with a negative control (ctrl) siRNA. Forty-eight hours p.t., cells were transfected with the plasmids required for a replication-competent minigenome assay. Forty-eight hours later, reporter activity was measured. (**B**) Analysis of viral transcription and protein expression in NXF1 knockdown cells in the absence of genome replication. 293T cells were transfected with siRNAs and 48 h p.t. with the plasmids required for a replication-deficient minigenome assay. After a further 48 h, reporter activity was analyzed. (**C**) Analysis of viral mRNA levels in NXF1 knockdown cells. Forty-eight hours after siRNA transfection of 293T cells, the cells were transfected with all components for the replication-competent minigenome assay. Forty-eight hours later RNA was isolated and RT-qPCR for viral mRNA was performed. (**D**) Analysis of viral gene expression after infection of NXF1 knockdown cells infected with recombinant EBOV expressing firefly luciferase. Reporter activity was determined one day p.i. and is shown relative to the reporter activity of cells treated with negative control siRNA. (**E**) Influence of NXF1 knockdown on viral mRNA synthesis. RNA from cells from panel **D** was isolated and luciferase mRNA was quantified using RT-qPCR. (**F**) Interaction of NXF1 with viral mRNA. 293T cells were transfected with plasmids encoding flag/HA-NXF1 or flag/HA-NXF1-mutants as indicated, in addition to the replication-competent minigenome, T7-polymerase, and the RNP proteins. As a control L was omitted (-L). Further negative controls (shown in grey) were as indicated. Forty-eight hours p.t., pulldowns were performed using anti-flag antibodies and RNA was isolated from the precipitates. mRNA levels in the precipitates were analyzed by subsequent RT-qPCR. (**G**) Interaction of NXF1 with viral genomic RNA. IP with anti-flag antibodies was performed with cell lysates from 293T cells transfected with plasmids encoding the replication-competent minigenome, T7-polymerase, and either flag/HA-NXF1, or flag/HA-NP, VP35, and VP24 (RNP). In controls the minigenome was omitted (-1cis). Further negative controls (shown in grey) were as indicated. RNA was isolated from the precipitates and analyzed via RT-qPCR. Means and standard deviations for two independent experiments (six biological replicates) are shown in panels **D** and **E**, and for three independent experiments in all other panels. Asterisks indicate *p* values from one-way ANOVA (*: *p* ≤ 0.05; **: *p* ≤ 0.01; ***: *p* ≤ 0.001; ****: *p* ≤ 0.0001; ns: *p* > 0.05).

As we had previously identified NXF1 in a genome-wide siRNA screen as being necessary for viral RNA synthesis and/or viral protein expression [19], we wanted to further dissect its impact on viral RNA synthesis and protein expression by assessing which of the different steps of these processes are influenced by NXF1. Therefore, we first performed classical EBOV minigenome assays in the context of an siRNA-mediated knockdown of endogenous NXF1. As shown before, the knockdown of NXF1 led to a reduction of reporter activity, confirming a role of NXF1 in viral RNA synthesis and/or gene expression (Figure 7A). In contrast to this, overexpression of NXF1 did not influence reporter activity (Figure S2), suggesting that the endogenous levels of NXF1 are sufficient to fulfill its function in the virus life cycle. To distinguish between a function of NXF1 in viral genome replication or in transcription/protein expression, we next used a replication-deficient minigenome, which lacks 55 nt in the trailer region, thus abolishing viral genome replication while still allowing transcription of the minigenome as previously described [41]. Using this minigenome system, NXF1 knockdown resulted in an 11.2 to 33.5-fold reduction in reporter activity, demonstrating that NXF1 is important for either viral transcription or protein expression, independent of viral genome replication (Figure 7B). In order to discriminate between an effect on viral mRNA transcription and later steps of viral protein expression such as mRNA transport from inclusion bodies or mRNA translation we next analyzed the influence of NXF1 knockdown on minigenome-derived viral mRNA levels. To this end, we again performed minigenome assays in NXF1 siRNA-treated cells, and analyzed the levels of viral mRNA in the cell lysates via RT-qPCR (Figure 7C). As the mRNA levels in the NXF1 knockdown cells were comparable to the control cells, NXF1 seems not to be important for viral transcription, but rather for either mRNA transport out of inclusion bodies towards ribosomes or efficient mRNA translation.

To confirm these findings in context of infectious EBOV, we repeated these experiments under BSL4 conditions using a recombinant EBOV that expresses firefly luciferase from an additional transcriptional unit, and thus allows precise quantification of viral gene expression [43]. In case of NXF1 knockdown we observed a significant reduction in luciferase activity (Figure 7D), and thus in viral gene expression compared to the negative control. In contrast, when we determined the amount of luciferase mRNAs (Figure 7E), there was no reduction but rather an increase in the mRNA levels upon NXF1 knockdown compared to the negative control, although this increase was not statistically significant. In contrast, knockdown of the viral polymerase L resulted in reductions in both reporter activity and mRNA levels by 62%. This indicates that also in context of a viral infection NXF1 is important for a late step in viral protein expression, i.e., either mRNA transport or efficient mRNA translation, but not for mRNA synthesis.

To further determine whether the influence of NXF1 on reporter protein expression can be explained by binding of viral mRNAs by NXF1, coIPs of viral mRNA in the context of a minigenome assay were performed (Figure 7F). To this end, NXF1 was precipitated and mRNA was isolated from the precipitate. When viral mRNA was produced (i.e., in the sample where the viral polymerase L was present; NXF1 +L in Figure 7F), we detected viral mRNA that could be co-precipitated together with NXF1. This was in contrast to the background signals observed in the control sample without polymerase (NXF1 -L) or in the negative control samples (-NXF1, mock IP, -RT). In contrast, the mutant lacking the RNA-binding domain (NXF1-ΔRBD) failed to co-precipitate the viral mRNA. However, coIP with NXF1-ΔRRM led to similar amounts of viral mRNA in the precipitate compared to wildtype NXF1. These results indicate that NXF1 interacts with the minigenome-derived viral mRNA, and that this interaction is mediated by the RBD of NXF1.

Finally, to check whether the interaction of NXF1 with the viral mRNA is specific for mRNA, or whether NXF1 binds viral RNA (vRNA) as well, we performed coIPs of minigenome vRNA together with NXF1 (Figure 7G). For this experiment the vRNA was generated by ectopically expressed T7 bacteriophage RNA polymerase in the absence of any viral proteins. As a positive control, vRNA was co-expressed with flag/HA-NP, VP35 and VP24, since these three proteins have been described as being minimally required for the formation of vRNA-containing nucleocapsids, and as such are necessary for co-precipitation of vRNA together with NP [7,48]. RNA was isolated from the precipitates and subsequent RT-qPCR showed that vRNA could indeed be co-precipitated with NP under these conditions (compare RNP +1cis vs. RNP -1cis in Figure 7G). In contrast, NXF1 failed to efficiently co-precipitate minigenome vRNA, suggesting a specific interaction with viral mRNAs.

### 3.6. A Model for the Function of NXF1 in the EBOV Life Cycle

**Figure 8:**
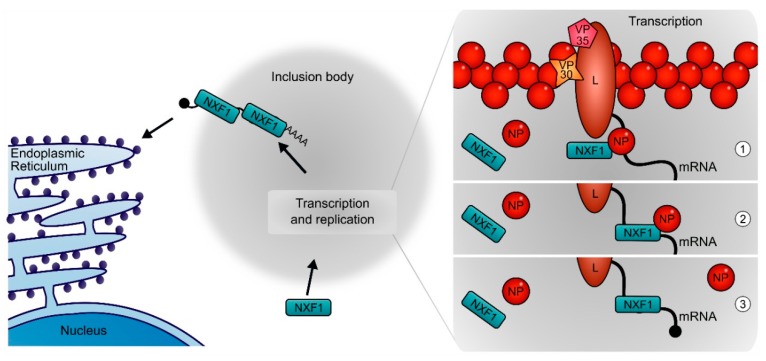
Model for the role of NXF1 and its interaction with NP and the viral mRNA during the EBOV life cycle. NXF1 enters inclusion bodies, which are the site of viral genome replication and transcription. Once in the inclusion bodies, NP recruits NXF1 to nascent mRNA during viral transcription (**1**). NXF1 then takes over the viral mRNA from NP (**2**), and thereby avoids encapsidation of the mRNA (**3**). After the completion of transcription, NXF1 together with the viral mRNA is exported from the inclusion bodies and transports it to ribosomes for translation.

Our results show that NXF1 is recruited to inclusion bodies, which we and others have shown to be sites of EBOV replication and transcription [10,14]. Furthermore, it is able to interact with NP, with the RBD of NXF1 being both necessary and sufficient for this interaction. In the context of its endogenous function, NXF1 has been shown to interact with many cellular proteins, including export adaptor proteins such as Aly and THOC5, to promote efficient mRNA binding (reviewed in [49]). Binding of these adaptor proteins, which are both components of the transcription and export complex (TREX), leads to a structural rearrangement of NXF1 [29,31]. This conformational change leads to exposure of the RBD resulting in an enhanced RNA-binding activity and subsequently to a handover of mRNA from Aly to NXF1. Since the interaction of NP with NXF1 resembles that of Aly and NXF1, as it involves the RBD, but not the amino acids that are important for the interaction with RNA [31], we propose a model in which a binding of NP to NXF1 results in a handover of viral mRNA from NP to NXF1 (Figure 8). Indeed, we were able to show that NXF1 binds mRNAs that were produced in inclusion bodies. We further propose that NXF1 then rapidly exports the bound RNA from inclusion bodies. This is in line with our observations that only RNA binding-deficient NXF1 accumulates in these structures, while wildtype NXF1 can only be observed in inclusion bodies in minute amounts, as well as the fact that the interaction of NP and NXF1 is weakened when NXF1 binds to RNA.

This model could explain how EBOV is able to overcome a number of fundamental challenges it faces during its life cycle, and which it shares with other cytoplasmically replicating NSVs. First, due to the RNA nature of its genome EBOV has to perform both genome replication and transcription, which exhibit fundamental differences: while both processes involve the generation of RNAs from a viral RNA template, during genome replication an accurate and complete copy of the RNA template has to be produced that is then encapsidated by NP to form a new RNP. This is in contrast to the subgenomic RNA fragments produced during transcription, which are processed by the addition of a cap structure and of a poly(A)-tail, and are not encapsidated by NP. While the production of subgenomic RNAs as well as their capping and poly(A)-tailing are all carried out by the viral polymerase L (reviewed in [50]), it is not clear how viral mRNAs evade encapsidation by NP, which is present in abundant quantities in inclusion bodies. However, co-transcriptional binding of viral mRNAs by NXF1 could shield the mRNA from such encapsidation by NP and/or displace already bound NP.

A second challenge during the EBOV life cycle is the fact that viral RNA synthesis occurs in inclusion bodies. These are increasingly appreciated for their importance in the genome replication of cytoplasmically replicating NSVs [12,21,22,23,51], and have for some of these viruses been described to show properties of liquid organelles [24,52]. Similar properties have been observed for EBOV inclusion bodies [10], suggesting that inclusion bodies of NSVs might generally represent liquid organelles. Such liquid organelles consist in most cases of RNA and RNA-binding proteins, and are formed by liquid-liquid phase separation, with multivalent low affinity interactions and intrinsically disordered protein regions being important drivers of their formation (reviewed in [53,54]). RNAs are important constituents of these liquid organelles and sometimes even the seed for their formation; however, in the case of mRNAs they have to be exported in order to reach ribosomes for translation. It is not clear how naked viral RNAs such as mRNAs would be able to escape this liquid organelle environment. To this end, their decoration with NXF1 might help viral mRNA to be exported from inclusion bodies by phase separation between the RNA:NXF1 complexes and other inclusion body constituents.

Finally, in contrast to most cellular mRNAs, the mRNAs of cytoplasmically replicating NSVs do not undergo splicing. Besides the removal of introns, splicing also leads to the association of mRNAs with a multitude of cellular proteins including NXF1, and has been shown to greatly improve translation from both cellular and viral mRNAs, independent of any effects on nuclear mRNA export [55,56,57]. Interestingly, gammaretroviruses usurp the NXF1 pathway to increase the translation efficiency of their unspliced mRNAs [40], and thus it is possible that the recruitment of NXF1 to EBOV mRNAs might serve a similar purpose.

Nevertheless, at this time point we cannot fully exclude that NXF1 fulfills another, not yet described function in the late stages of viral protein expression, and future studies will be required to prove or disprove the model proposed by us. Further, it will be interesting to dissect mechanistic details of the proposed export of viral mRNAs from inclusion bodies, as well as the precise localization of NXF1 in inclusion bodies, and specifically whether there is a compartmentalization of different aspects of the virus life cycle inside inclusion bodies that correlates with the localization of NXF1 and other host factors. Finally, it is intriguing that when we first identified NXF1 as a factor involved in viral RNA synthesis and/or protein expression, we could show that it is also important for these processes for Junín virus, a viral hemorrhagic fever-causing arenavirus [19] that, like EBOV, performs its genome replication in inclusion bodies [21]. It will be interesting to see whether NXF1 fulfills the same role in the arenavirus life cycle as in the filovirus life cycle, and given that the fundamental nature of the challenges described above are shared between many if not all cytoplasmically replicating negative-sense RNA viruses, whether other such viruses also are dependent on NXF1.

## Supplementary Materials

The following are available online at https://www.mdpi.com/2073-4409/9/1/187/s1, Figure S1: RNA dependence of the interaction between NXF1 and VP35; Figure S2: Influence of NXF1 overexpression on viral RNA synthesis and protein expression.
