# Supplementary material for: The Ebola Virus Nucleoprotein Recruits the Nuclear RNA Export Factor NXF1 into Inclusion Bodies to Facilitate Viral Protein Expression
Source: Cells. 2020 Jan 11;9(1):187. doi: 10.3390/cells9010187 (PMC7017048; doi:10.3390/cells9010187)
Supplement: Supplementary file 1 [file cells-09-00187-s001.pdf]

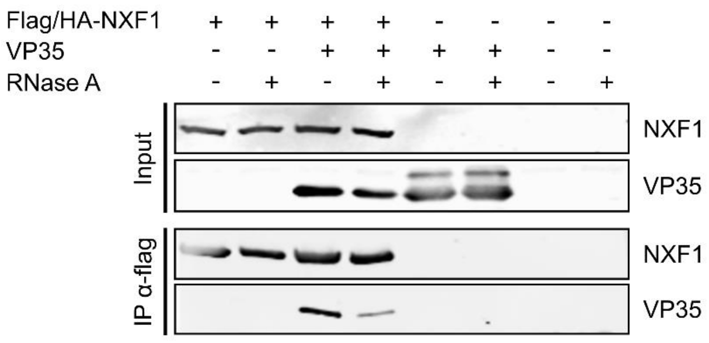

Figure S1. RNA dependence of the interaction between NXF1 and VP35. 293T cells were transfected with plasmids encoding for flag/HA-NXF1 and VP35. Forty-eight hours post transfection, cells were lysed and were either treated with RNase A (100 µg/ml) or remained untreated before samples were subjected to immunoprecipitation with anti-flag antibodies. Input and precipitates were analyzed via SDS-PAGE and Western blot using anti-flag and anti-VP35 antibodies

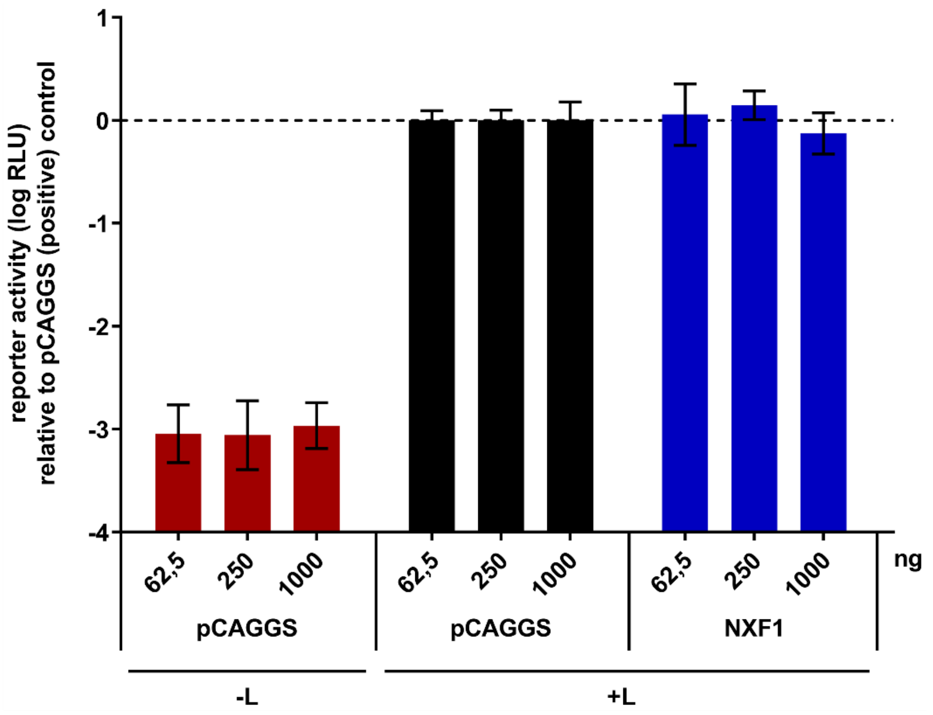

Figure S2. Influence of NXF1 overexpression on viral RNA synthesis and protein expression. 293T cells were transfected with plasmids encoding for all minigenome components (minigenome, NP, VP35, VP30, polymerase L, T7) as well as either NXF1 or empty vector (pCAGGS). As negative control the plasmid encoding for the viral polymerase was omitted. Reporter activities in relation to cells transfected with empty vector are shown.
